# Effects of Widespread Inotrope Use in Acute Heart Failure Patients

**DOI:** 10.3390/jcm7100368

**Published:** 2018-10-18

**Authors:** Jeehoon Kang, Hyun-Jai Cho, Hae-Young Lee, Sangjun Lee, Sue K. Park, Sang Eun Lee, Jae-Joong Kim, Eun-Seok Jeon, Shung Chull Chae, Sang Hong Baek, Seok-Min Kang, Dong-Ju Choi, Byung-Su Yoo, Kye Hun Kim, Myeong-Chan Cho, Byung-Hee Oh

**Affiliations:** 1 Department of Internal Medicine, Seoul National University Hospital, Seoul 03080, Korea; medikang@gmail.com (J.K.); hylee612@snu.ac.kr (H.-Y.L.); ohbhmed@snu.ac.kr (B.-H.O.); 2 Department of Preventive Medicine and Department of Biomedical Science, Seoul National University College of Medicine, Seoul 03080, Korea; sjunlee@snu.ac.kr (S.L.); suepark@snu.ac.kr (S.K.P.); 3 Department of Cardiology, Asian Medical Center, Seoul 05505, Korea; sangeunlee.md@gmail.com (S.E.L.); jjkim@amc.seoul.kr (J.-J.K.); 4 Department of Medicine, Sungkyunkwan University College of Medicine, Seoul 16419, Korea; eunseok.jeon@samsung.com; 5 Department of Internal Medicine, Kyungpook National University College of Medicine, Daegu 37224, Korea; scchae@knu.ac.kr; 6 Department of Internal Medicine, The Catholic University of Korea, Seoul 06591, Korea; whitesh@catholic.ac.kr; 7 Yonsei University College of Medicine, Seoul 03722, Korea; smkang@yumc.yonsei.ac.kr; 8 Department of Internal Medicine, Seoul National University Bundang Hospital, Seongnam 13620, Korea; djchoi@snu.ac.kr; 9 Department of Internal Medicine, Yonsei University Wonju College of Medicine, Wonju 26426, Korea; yubs@yonsei.ac.kr; 10 Heart Research Center of Chonnam National University, Gwangju 61469, Korea; christiankyehun@hanmail.net; 11 Department of Internal Medicine, Chungbuk National University College of Medicine, Cheongju 28644, Korea; mccho@chungbuk.ac.kr

**Keywords:** acute heart failure, inotrope, initial systolic, blood pressure

## Abstract

Current guidelines recommend that inotropes should not be used in patients with normal systolic blood pressure (SBP). However, this is not supported with concrete evidence. We aimed to evaluate the effect of inotropes in acute heart failure (HF) patients from a nationwide HF registry. A total of 5625 patients from the Korean Acute Heart Failure (KorAHF) registry were analyzed. The primary outcomes were in-hospital adverse events and 1-month mortality. Among the total population, 1703 (31.1%) received inotropes during admission. Inotrope users had a higher event rate than non-users (in-hospital adverse events: 13.3% vs. 1.4%, *p* < 0.001; 1-month mortality: 5.5% vs. 2.5%, *p* < 0.001), while inotrope use was an independent predictor for clinical outcomes (in-hospital adverse events: OR_adjusted_ 5.459, 95% CI 3.622–8.227, *p* < 0.001; 1-month mortality: HR_adjusted_ 1.839, 95% CI 1.227–2.757, *p* = 0.003). Subgroup analysis showed that inotrope use was an independent predictor for detrimental outcomes only in patients with normal initial SBP (≥90 mmHg) (in-hospital adverse events: OR_adjusted_ 5.931, 95% CI 3.864–9.104, *p* < 0.001; 1-month mortality: HR_adjusted_ 3.584, 95% CI 1.280–10.037, *p* = 0.015), and a propensity score-matched population showed consistent results. Clinicians should be cautious with the usage of inotropes in acute heart failure patients, especially in those with a normal SBP.

## 1. Introduction

Acute heart failure (HF), defined as a rapid onset of, or change in, symptoms and signs of HF can be a life-threatening condition that requires immediate medical attention [1,2]. Acute HF is itself one of the leading causes of hospitalization and usually leads to hospitalization, which is associated with substantial mortality and morbidity [3]. Generally, acute HF occurs following a precipitating factor such as excessive fluid or salt intake, medication non-adherence, myocardial infarction, or arrhythmia, or concurrent non-cardiac illness (e.g., infection, embolism, thyroid disease, renal failure, etc.) [4]. The overall goal of treating acute HF is focused on identifying any precipitating factors, relieving symptoms, and optimizing long-term therapies. However, despite the vast number of trials investigating optimal treatment for chronic HF, treatment guidelines for acute HF are still not well established.

The majority of acute HF patients are treated with diuretics to optimize volume status and vasodilators for symptom resolution and congestion relief. Inotropes can be administered in patients with hypotension or signs and symptoms of peripheral hypo-perfusion. However, previous clinical trials have failed to show any benefit from inotropes, even in symptom relief [5,6,7]. Meanwhile, the 2016 European guidelines confirm inotrope use as a level of recommendation of class III (not to be used) for patients with normal systolic blood pressure (SBP) >90 mmHg, and without cardiogenic shock. The evidence supporting this recommendation is quite weak, only derived from retrospective studies or meta-analyses, which has led to a decrease in adherence to guideline-based therapy. Therefore, the aim of our study was to support the present guideline by evaluating the effect of inotropes in acute HF patients from a prospective nationwide HF registry. From our acute HF registry, we comprehensively evaluated the safety of inotrope use during admission and for a follow-up period.

## 2. Materials and Methods

The KorAHF (Korean Acute Heart Failure) registry is a prospective multicenter cohort study that was designed to describe patient characteristics, current treatments, and short- and long-term patient outcomes among Korean patients with acute HF (NCT01389843). A total of 5625 patients were enrolled from the KorAHF registry between March 2011 and February 2014 at 10 tertiary centers. The rationale and detailed study design of the KorAHF registry has been presented in previous publications [8]. Briefly, patients with signs or symptoms of HF and either lung congestion, objective findings of LV systolic dysfunction, or structural heart disease were eligible for the study. Detailed variables were collected at baseline admission, and events including all-cause mortality, mortality from HF aggravation, and re-hospitalization for HF aggravation were recorded after discharge. Follow-up data up to 60 months after discharge were collected from the patients by the attending physician.

The study protocol was approved by the ethics committee at each participating center, and was conducted according to the principles of the Declaration of Helsinki. All patients provided written informed consent for participation in the registry.

### 2.1. Study Endpoints and Definitions

The clinical outcomes included adverse in-hospital outcomes (defined as all-cause mortality or aggravation of HF during hospitalization) and all-cause mortality at one month after discharge. In-hospital mortalities and the causes of death were adjudicated by an independent event committee. The definition of each category is described in the Supplementary method. This adjudication form was referenced by the adjudication of death of typical 3-phase randomized control trials such as the RELAX and RELAX-AHF-2 trial [9,10].

The mortality data for patients who were lost to follow-up were collected from the National Insurance data or National Death Records.

Inotropes included dobutamine, dopamine, norepinephrine, and vasopressin, while inotrope use was defined as at least one use of an inotropic agent, regardless of the cause. The initial SBP was defined as the initial blood pressure measured immediately after admission. Left ventricular ejection fraction (LVEF) values were obtained by transthoracic echocardiography performed during the index hospitalization. Quantitative calculation using the modified Simpson’s biplane method was recommended for LVEF measurement, but visually estimated LVEF was also accepted as valid for HF categorization. According to the 2016 ESC HF guidelines, heart failure with preserved ejection fraction (HFpEF) was defined as LVEF ≥ 50% and heart failure with reduced ejection fraction (HFrEF) was defined as LVEF < 40%. Subjects missing quantitative LVEF data were excluded from the final analysis.

### 2.2. Statistical Analyses

All variables and outcome analyses were based on inotrope use. Data were presented as numbers and frequencies for categorical variables and as means ± standard deviation (SD) for continuous variables. For comparison among groups, the χ^2^ test (or Fisher’s exact test when any expected count was <5 for a 2 × 2 table) for categorical variables and unpaired Student’s t-test or one-way analysis of variance for continuous variables were applied.

To estimate the predictors of inotrope use and to predict the independent effects of inotrope use on adverse in-hospital outcomes, we used a multivariable logistic regression model using a stepwise algorithm. Variables found to be statistically significant in the univariate analysis were included in the multivariable model, excluding variables that were closely related to other clinical variables. As a result, variables such as sex, age, body mass index, previous hypertension, previous diabetes mellitus, previous chronic renal failure, previous percutaneous intervention, previous myocardial infarction, atrial fibrillation, initial SBP, initial New York Heart Association (NYHA) classification, LVEF value, vasodilator use, renal replacement therapy during admission, and lab tests such as sodium, hemoglobin, uric acid, C-reactive protein, and natriuretic peptides (B type natriuretic peptide or N-terminal pro-B type natriuretic peptide) were included in the model. Assumptions of the logistic regression model (e.g., dichotomous dependent variable, independence in each observation, linear relationship between continuous independent variables and the logit transformation of the dependent variable, and multi-collinearity) were tested. The Cox and Snell R Square and Hosmer–Lemeshow goodness-of-fit test were used to evaluate model calibration.

Given the difference in baseline characteristics between groups, we used two different matching processes, the propensity score matching (PSM) method and the inverse probability weighted (IPW) Cox proportional hazards regression model. For the PSM analysis, we performed 1:1 matching for inotropic users and non-users. A logistic regression model was conducted to generate the propensity score, which was the probability that a patient received inotropes. The same variables mentioned in the preceding paragraph were considered as adjusted covariates to calculate for the propensity score. Then, a greedy matching algorithm was used to match patients on the logit of the propensity score with a caliper width of 0.5 of the SD of the logit of the propensity score. Baseline clinical characteristics and laboratory findings were compared within the propensity score matched group. The statistical significance for propensity-score matched groups was assessed by a paired t-test or Wilcoxon signed rank test, or extensions thereof for categorical variables with more than two levels. The success of the propensity score was estimated by assessing the balance of baseline characteristics after propensity-score matching. The balance of each variable between the two groups was evaluated by the standardized mean difference. The identical propensity score was also used in for the IPW Cox proportional hazards regression model to adjust for uneven distribution of baseline characteristics between the different groups. C-statistics with 95% confidence intervals (CIs) were calculated to validate the discriminant function of the model.

A two-sided probability value less than 0.05 was considered to estimate statistically significant differences. Statistical tests were performed using SPSS v22.0 (IMB Corporation, Armonk, NY, USA) and Stata, version 10 (2007, Stata Corporation, College Station, TX, USA).

## 3. Results

**Figure 1:**
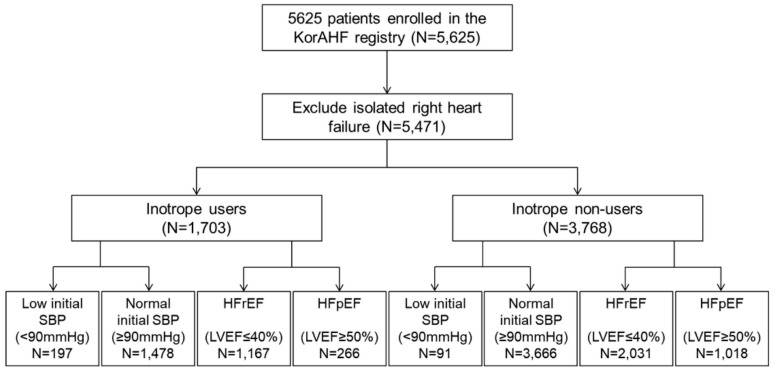
Flowchart of the Korean Acute Heart Failure (KorAHF) registry. Analysis was performed to evaluate the effect of inotropes in acute heart failure patients. Subgroup analysis was performed by initial SBP (low initial SBP (<90 mmHg) vs. normal initial SBP (≥90 mmHg)) LV systolic function (HFrEF (LVEF ≤ 40%) vs. HFpEF (LVEF ≥ 50%)).

A total of 5625 patients from the KorAHF registry were analyzed. Those with isolated right HF (154 patients, 2.7%) were excluded, leaving 1703 (31.1%) patients who received inotropes during admission and 3768 (68.6%) patients who did not receive inotropes (Figure 1). Table 1 shows the baseline characteristics, while ischemic heart disease was the main underlying etiology of acute HF was ischemic heart disease (Table S1). Patients who received inotropes had a different risk factor profile (Table 1), and a distinct medication prescription pattern (Table S2). Among inotrope users 44.7% used multiple inotropic agents, and the mean number of inotropic agent was 1.68 ± 0.88 per patient (Table S3). For the subgroup analysis, patients were divided into those with a low initial SBP (<90 mmHg) vs. normal initial SBP (≥90 mmHg) and those with HFrEF vs. HFpEF. Use of inotropes was more frequent in patients with a low initial SBP than in those with a normal initial SBP (68.4% [197/288] vs. 28.7% [1478/5144], *p* < 0.001) and in HFrEF patients than in HFpEF patients (50.3% [929/1848] vs. 33.6% [237/705], *p* < 0.001). Interestingly, 19.9% (247/1239) of HFpEF patients with a normal SBP received inotropes. 

### 3.1. In-Hospital Clinical Events

In-hospital clinical events occurred in 280 (5.1%) of the cases during admission. Patients who were given inotropes had a higher rate of in-hospital clinical events (227/1703 (13.3%) vs. 53/3768 (1.4%), *p* < 0.001), which was consistent with the subgroup analysis (Table 2). Cardiac death occupied the majority of in-hospital clinical events (70.3%), and the most common cause of cardiac death was HF aggravation (60.4%; Figure S1). In subgroup analysis, cardiac deaths were more common in inotrope users compared with non-users (76.2% (173/227) vs. 45.3% (24/53), *p* < 0.001). Importantly, all eight cases of in-hospital sudden cardiac death, which was defined as death that occurred unexpectedly in clinically stabilized patients, occurred in inotrope users, composing 4.6% of total cardiac death events. In contrast, non-cardiovascular deaths were more common in non-users than users (24.5% (13/53) vs. 13.7% (31/227), *p* = 0.050).

The use of inotropes was an independent predictor of in-hospital clinical events (OR_adjusted_ 5.459, 95% CI 3.622–8.227, *p* < 0.001), whereas other independent predictors included factors such as old age, low body mass index, baseline chronic renal failure, HFrEF, high uric acid level, high C-reactive protein, and renal replacement therapy during admission (Table 3). When divided into subgroups by the initial SBP, use of inotropes was only a risk factor in patients with an initial SBP ≥ 90 mmHg (OR_adjusted_ 5.931, 95% CI 3.864–9.104, *p* < 0.001). According to the LV systolic function, inotrope use was an independent risk factor in both HFrEF (OR_adjusted_ 5.699, 95% CI 3.529–9.205, *p* < 0.001) and HFpEF (OR_adjusted_ 4.374, 95% CI 1.476–12.962, *p* = 0.008) (Table S4). There were no significant interactions across subgroups by the initial SBP (*p* = 0.133) and LV systolic function (*p* = 0.999).

### 3.2. Post-Discharge 1-Month Mortality

**Figure 2:**
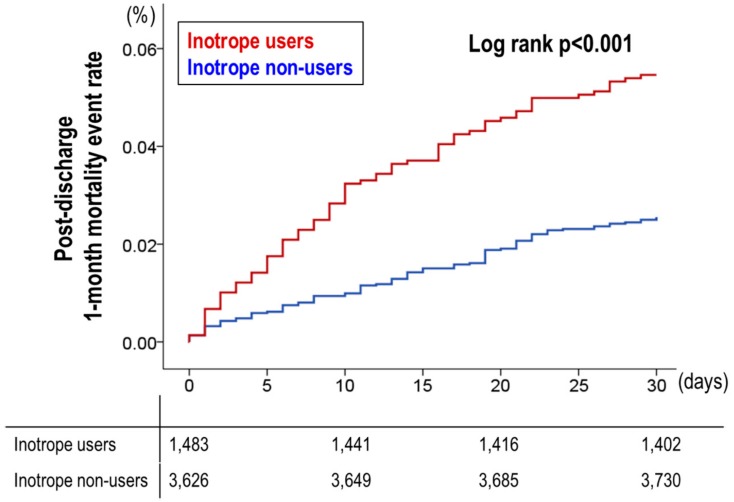
Post-discharge 1-month mortality events by inotrope use. The post-discharge 1-month survival curve showed that the post-discharge 1-month mortality was significantly different between inotrope users and non-users.

At 1 month after discharge, 3.4% (176/5213) of patients expired, among which inotrope users had a significantly higher rate of post-discharge 1-month mortality (5.5% (81/1483) vs. 2.5% (95/3730), *p* < 0.001) (Table 4). The post-discharge medications are presented in Table S5. Inotrope use was an independent predictor of post-discharge 1-month mortality with a HR_adjusted_ of 1.839 (95% CI 1.227–2.757, *p* = 0.003), while old age, hyponatremia, renal replacement therapy during admission, high uric acid, and high c-reactive protein were the associated independent predictors (Table 5). As shown in Figure 2, the post-discharge 1-month survival curve also showed that the 1-month mortality after discharge was significantly different between inotrope users and non-users.

**Figure 3:**
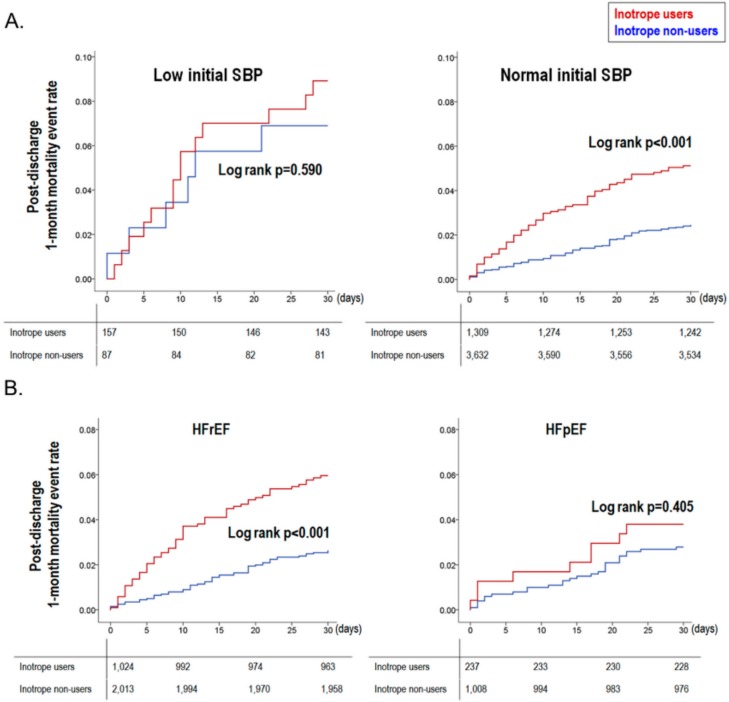
Post-discharge 1-month mortality events by inotrope use in subgroup analysis. The post-discharge 1-month survival curves showed that (**A**) mortality evens occurred more often in inotrope users only in patients with a normal initial SBP (≥90 mmHg), and (**B**) in patients with a depressed LV systolic function (LVEF ≤ 40%).

In the subgroup analysis by the initial SBP and by LV systolic function, inotrope use had a distinct effect size according to each subgroup. Inotrope use was an independent predictor of post-discharge 1-month mortality in those with a normal initial SBP (HR_adjusted_ 3.584, 95% CI 1.280–10.037, *p* = 0.015) and in those with HFpEF (HR_adjusted_ 54.666, 95% CI 3.479–858.978, *p* = 0.004, Table S6). The survival curves showed consistent results, as shown in Figure 3. However, no significant interactions were observed across subgroups by both initial SBP (*p* = 0.343) and LV systolic function (*p* = 0.224).

### 3.3. Propensity Score Matched Population

Because of the distinct baseline characteristics among inotrope users and non-users, we performed two different methods to compensate for the differences: the PSM method and the IPW Cox proportional hazards regression model. By using the PSM method, 989 pairs of inotrope users and non-users were matched. Baseline characteristics showed a decreased difference between the two groups. Among the PSM population, 114 (5.8%) patients presented with an initial SBP < 90 mmHg, while the proportion of inotrope users was similar to that among patients presenting with normal initial SBP (51.8% (59/114) vs. 49.9% (930/1864), *p* = 0.700). When we compared patients using the PSM method between normal and low SBP, patients with a low initial SBP were younger, had a lower body mass index, and had lesser cardiovascular risk factors, such as hypertension, diabetes mellitus, and valvular diseases than patients with a normal initial SBP (Table S7).

The crude rate of in-hospital adverse outcomes (7.9% (78/989) vs. 1.9% (19/989), *p* < 0.001) and post-discharge 1-month mortality (4.2% (39/921) vs. 2.6% (25/989), *p* = 0.045) was significantly higher in inotrope users (Table 6). The multivariable regression model showed that inotrope use was a significant predictor of in-hospital adverse events (OR_adjusted_ 3.893, 95% CI 2.289–6.621, *p* < 0.001), and in the subgroup analysis, inotrope use was an independent predictor only in those with a normal initial SBP (OR_adjusted_ 4.113, 95% CI 2.321–10.897, *p* < 0.001). Moreover, regarding the post-discharge 1-month clinical follow-up, inotrope use was a marginally significant predictor for post-discharge 1-month mortality (HR_adjusted_ 1.618, 95% CI 0.971–2.697, *p* = 0.065), while in those with a normal initial SBP, inotrope use was an independent predictor for post-discharge 1-month mortality (HR_adjusted_ 1.768, 95% CI 1.047–2.986, *p* = 0.033). We also performed an additional PSM analysis, by extreme strict matching that left 238 pairs in each population (Table S8). When the difference of baseline characteristics was almost indistinguishable between inotrope users and non-users, despite the small sample size, we could find that the trend was identical to the original analysis; adverse events occurred more commonly inotrope users.

The IPW Cox proportional hazards regression model also showed that inotrope use was a significant predictor of in-hospital adverse clinical outcomes (OR_adjusted_ 1.892, 95% CI 1.285–2.767, *p* = 0.001) and post-discharge 1-month mortality (HR_adjusted_ 1.870, 95% CI 1.285–2.721, *p* = 0.001). The subgroup analysis showed that inotrope use was an independent predictor for in-hospital adverse clinical outcomes and post-discharge 1-month mortality only in patients with a normal initial SBP (in-hospital adverse clinical outcome: OR_adjusted_ 2.924, 95% CI 1.157–7.474, *p* = 0.021; post-discharge 1-month mortality: HR_adjusted_ 1.829, 95% CI 1.227–2.726, *p* = 0.003). For patients with a low initial SBP, inotrope use was not a risk factor for adverse outcomes (in-hospital adverse clinical outcome: OR_adjusted_ 0.575, 95% CI 0.013–25.167, *p* = 0.730; post-discharge 1-month mortality: HR_adjusted_ 1.714, 95% CI 0.372–7.893, *p* = 0.489).

## 4. Discussion

Our study provides an important insight into the use of inotropes in patients admitted for acute HF. Of the total population, 31.1% (1703 patients) were inotrope users, among which 88.2% (1478 patients) had a normal initial SBP (SBP ≥ 90 mmHg). In-hospital adverse clinical events occurred in 5.1% (280 patients), and during the post-discharge 1-month follow-up period, 3.4% (176 patients) of the total population expired. Inotrope use was an independent predictor for in-hospital adverse clinical events and post-discharge 1-month mortality. Subgroup analysis revealed that inotrope use had a different effect size according to the initial SBP and LV systolic function, without significant interaction.

### 4.1. Inotrope Use in Acute HF

Acute HF is a medical condition that requires immediate medical management, and usually, urgent hospital admission [11]. The management of acute HF includes rapid recognition of the precipitant cause, initiation of specific treatment, and prompt symptom control. This includes diuretics for patients with signs of fluid overload and congestion, vasodilators for symptomatic relief, oxygen for hypoxemic patients, and thromboembolism prophylaxis [1,2]. In particular, patients with cardiogenic shock (defined as SBP < 90 mmHg) should receive immediate comprehensive assessment. When choosing pharmacologic agents to restore organ perfusion by increasing cardiac output and blood pressure, inotropic agents and vasopressors can be considered, along with device therapies such as intra-aortic balloon pumps [1]. Although loop diuretics are an essential component of therapy for acute HF, the limited evidence of previous studies give diuretics a class I recommendation, but based on level B or level C evidence [12]. Furthermore, the Diuretic Optimization Strategies Evaluation trial showed that high-dose diuretics might be associated with worsening renal function [13]. The lack of demonstrated benefit for high-dose diuretics may be related to stimulation of the renin–angiotensin–aldosterone and sympathetic nervous systems [14].

Accordingly, current guidelines recommend that inotropes, which are sympathetic stimulators, should be reserved for patients with a severe reduction in cardiac output, resulting in compromised organ perfusion. In particular, inotropic agents are not recommended before other potentially correctable factors are present. This is because of the concern for increased adverse effects by sympathetic stimulators, which is supported by previous studies [15,16].

Despite the negative implications of inotrope use from these studies, almost one-third (31.3%) of our study population received inotropes. Previous studies have also shown similar rates of inotrope use in HF patients. In the Italian Network on Heart Failure (IN-HF) outcome registry, 20% received inotropes [17], and in the Acute Heart Failure Global Survey of Standard Treatment (ALARM-HF) registry, 33% received inotropes [7]. Interestingly, only a small proportion of inotrope users were presented with low perfusion or cardiogenic shock (12.2%), elucidating the inappropriate use of inotropes in real-world practice.

### 4.2. Hazardous Effect of Inotropes in Acute HF Patients

From our study results, we have demonstrated the hazardous effects of inotrope use in acute HF patients from a nationwide HF registry. Inotrope use was an independent predictor of in-hospital adverse outcomes and 1-month mortality. Interestingly, in the subgroup analysis, inotrope use was only an independent predictor in patients with a normal blood pressure, while inotrope use had a neutral effect in those with systemic hypotension (SBP < 90 mmHg), even after compensating baseline risk factors and the different medication prescription patterns in each group. Based on the negative interaction across subgroups, we can assume that the initial SBP was not a dominant factor in determining the effect of inotropes, that is, a low initial SBP does not warrant a beneficial or neutral effect by inotropes. On the contrary, we can assume that a certain subgroup of patients within those with a low initial SBP may benefit from inotrope use. However, according to the LV systolic function, inotrope use was a significant predictor of in-hospital adverse outcomes in both HFrEF and HFpEF.

Our results are in line with current guidelines about the use of inotropic agents in the pharmacological treatment of acute HF. Inotropic agents are not recommended unless the patient has symptomatic hypotension or is hypoperfused, because of concerns about the safety of inotrope use (Class III, Level of Evidence A) [18]. In our results, inotrope use was hazardous in patients without initial hypotension. Meanwhile, guidelines note that short-term inotropes can be considered in patients with hypotension to maintain peripheral organ perfusion and function (Class IIb, Level of Evidence C) [1]. Because inotrope use showed deleterious effects on outcomes in our study, we can assume that inotropes may be used in very selective cases to improve peripheral hypoperfusion. Despite the main effect of inotropes and vasopressors, which is to increase cardiac contractility and cardiac output, and to restore blood pressure, previous data have failed to demonstrate the benefit with these agents [15,16,17,19]. This may be related to the deleterious effects of catecholamine stimulation in HF [20]. In contrast, beta-adrenergic blockers have been shown to reduce mortality and morbidity in symptomatic HF patients by blocking the effect of catecholamines. By counteracting the effect of catecholamines, beta-blockers have various effects, including reduction in heart rate, improvement of left ventricular remodeling, increase in LVEF, reduction in end-systolic volume, and maintaining anti-arrhythmic action [21,22]. However, this does not deny the potential beneficial effects of inotropes in selected cases. In patients with systemic hypotension and/or signs of hypoperfusion and shock, inotropes may be a breakthrough method to overcome the imminent unstable condition. 

### 4.3. Study Limitations

There are several limitations to this study. First, because patients with more severe clinical presentations are more likely to receive inotropes, inotrope use itself may be associated with worse clinical outcomes. This was observed in the distinct baseline characteristics between inotrope users and non-users. We performed various statistical methods to overcome the baseline differences; however, the possibility that confounding factors may have influenced our study results cannot be ruled out. Second, the exact dose and duration of inotropes were not analyzed because this was not available in our dataset. Future studies, which might provide a dose-dependent relationship between adverse effects and inotrope dosage, could support our study result. Third, regarding the nature of our study (a retrospective analysis of a prospective cohort), our study results are at best hypothesis-generating. However, because it is practically very difficult to demonstrate our study results in a randomized clinical trial, we believe that our study results should be taken with values regarding treatment strategies for acute HF patients in the clinical setting.

## 5. Conclusions

Inotropes are still widely used, even in acute HF patients presenting with a normal blood pressure. The infusion of inotropes is strongly associated with in-hospital adverse outcomes and 1-month follow-up mortalities. We should be cautious of inotrope use in acute HF patients.

## Figures and Tables

**Table 1:** Demographic and laboratory characteristics between inotrope users and inotrope non-users.

	Total Population (*n* = 5471)	Inotrope Users (*n* = 1703)	Inotrope Non-Users (*n* = 3768)	*p* Value
**Sex (male)**	2919 (53.4%)	1024 (60.1%)	1895 (50.3%)	<0.001
**Age (years old)**	68.6 ± 14.4	66.8 ± 14.8	69.4 ± 14.2	<0.001
**BMI (kg/m^2^)**	23.3 ± 3.9	22.9 ± 3.8	23.5 ± 3.9	<0.001
**LVEF (%)**	37.3 ± 15.5	33.0 ± 14.6	39.2 ± 15.5	<0.001
**Risk factors**				
**HTN, *n* (%)**	2211 (40.4%)	791 (46.4%)	1420 (37.7%)	<0.001
**DM, *n* (%)**	3518 (64.3%)	1053 (61.8%)	2465 (65.4%)	0.010
Smoking, % *	17.9/21.1/61.1	20.3/21.8/57.9	16.8/20.7/62.5	0.002
**Previous MI, *n* (%)**	909 (16.6%)	308 (18.1%)	601 (16.0%)	0.051
**Previous PCI, *n* (%)**	931 (17.0%)	318 (18.7%)	613 (16.3%)	0.030
**Previous CABG, *n* (%)**	287 (5.2%)	113 (6.6%)	174 (4.6%)	0.002
**COPD, *n* (%)**	602 (11.0%)	199 (11.7%)	403 (10.7%)	0.280
**CRF, *n* (%)**	791 (14.5%)	277 (16.3%)	514 (13.6%)	0.011
**Previous CVA, *n* (%)**	833 (15.2%)	237 (13.9%)	596 (15.8%)	0.069
**Valve disease, *n* (%)**	769 (14.1%)	295 (17.3%)	474 (12.6%)	<0.001
**Atrial fibrillation, *n* (%)**	1850 (33.8%)	465 (27.3%)	1385 (36.8%)	<0.001
**Heart failure Etiology**				
**Ischemic heart disease**	2096 (38.3%)	768 (45.1%)	1328 (35.2%)	<0.001
**Valvular heart disease**	763 (13.9%)	289 (17.0%)	474 (12.6%)	<0.001
**Congenital heart disease**	50 (0.9%)	17 (1.0%)	33 (0.9%)	0.659
**Cardiomyopathy**	1163 (21.3%)	374 (22.0%)	789 (20.9%)	0.392
**Hypertension**	216 (3.9%)	24 (1.4%)	192 (5.1%)	<0.001
**Myocarditis**	75 (1.4%)	44 (2.6%)	31 (0.8%)	<0.001
**Infiltrative disease ^†^**	69 (1.3%)	13 (0.8%)	56 (1.5%)	0.027
**Tachycardia related disease ^#^**	586 (10.7%)	69 (4.1%)	517 (13.7%)	<0.001
**Thyroid related disease ^$^**	29 (0.5%)	4 (0.2%)	25 (0.7%)	0.043
**Toxic related disease** **^‡^**	58 (1.1%)	19 (1.1%)	39 (1.0%)	0.787
NYHA at admission, (%) **	15.2/36.6/48.2	11.1/33.0/55.9	17.1/38.2/44.7	<0.001
**Initial SBP (mmHg)**	131 ± 30	121 ± 30	136 ± 30	<0.001
**Initial DBP (mmHg)**	79 ± 19	74 ± 18	81 ± 19	<0.001
**Initial HR**	93 ± 26	93 ± 25	93 ± 26	0.432
**Laboratory analysis**				
**WBC (10^9^/L)**	8710 ± 3960	9480 ± 4620	8360 ± 3560	<0.001
**Hb (g/dL)**	12.4 ± 3.0	12.4 ± 2.3	12.4 ± 2.3	0.465
**Platelet (10^9^/L)**	211 ± 89	204 ± 89	215 ± 88	<0.001
**Total cholesterol (mg/dL)**	152 ± 43	148 ± 43	154 ± 43	<0.001
**Triglyceride (mg/dL)**	100 ± 59	98 ± 64	101 ± 57	0.193
**HDL (mg/dL)**	41 ± 14	39 ± 14	42 ± 14	<0.001
**LDL (mg/dL)**	107 ± 55	104 ± 56	108 ± 55	0.059
**Na (mEq/L)**	138 ± 5	137 ± 5	138 ± 4	<0.001
**Uric acid (mg/dL)**	7.0 ± 2.9	7.4 ± 3.0	6.9 ± 2.8	<0.001
**BUN (mg/dL)**	26 ± 16	29 ± 19	25 ± 15	<0.001
**Creatinine (mg/dL)**	1.49 ± 1.49	1.62 ± 1.60	1.43 ± 1.43	<0.001
**Glucose (mg/dL)**	156 ± 77	166 ± 86	151 ± 73	<0.001
**CRP (mg/L)**	2.36 ± 4.25	3.15 ± 5.33	2.00 ± 3.60	<0.001
**BNP (pg/mL)**	1341 ± 1304	1571 ± 1454	1243 ± 1223	<0.001
**NTproBNP (pg/mL)**	9327 ± 10846	11762 ± 12089	8232 ± 10051	<0.001

BMI, body mass index; LVEF, left ventricular ejection fraction; HTN, hypertension; DM, diabetes mellitus; MI, myocardial infarction; PCI, percutaneous coronary intervention; CABG, coronary artery bypass graft surgery; COPD, chronic obstructive pulmonary disease; CRF, chronic renal failure; CVA, cerebrovascular accident; SBP, systolic blood pressure; DBP, diastolic blood pressure; HR, heart rate; WBC, white blood cell; Hb, hemoglobin; HDL, high density lipoprotein; LDL, low density lipoprotein, Na; sodium; BUN, blood urea nitrogen; CRP, C-reactive protein; BUN, brain natriuretic peptide; NTproBNP, n-terminal brain natriuretic peptide. * smoking: current smoker/ex-smoker/never smoked. ^†^ Infiltrative heart disease includes amyloidosis and sarcoidosis. ^#^ Tachycardia-related disease mainly includes atrial fibrillation, Atrial tachycardia and ventricular tachycardia. ^$^ Thyroid-related disease includes hyperthyroidism and hypothyroidism. ^‡^ Toxins of toxic related disease include antineoplastic drugs, heavy metals, and alcohol. ** NYHA: grade 2/grade 3/grade 4.

**Table 2:** In hospital clinical event rate. HFpEF, heart failure with preserved ejection fraction; HFrEF, heart failure with reduced ejection fraction.

	Total	Inotrope Users	Inotrope Non-Users	*p* Value
**Total population**				
**Primary endpoint**	280/5471 (5.1%)	227/1703 (13.3%)	53/3768 (1.4%)	<0.001
**Mortality**	258/5471 (4.7%)	220/1703 (12.9%)	38/3768 (1.0%)	<0.001
**Cardiac death**	197/5471 (3.6%)	173/1703 (10.2%)	24/3768 (0.6%)	<0.001
**Normal initial SBP (SBP ≥ 90 mmHg)**				
**Primary endpoint**	225/5144 (4.4%)	176/1478 (11.9%)	49/3666 (1.3%)	<0.001
**Mortality**	203/5144 (3.9%)	169/1478 (11.4%)	34/3666 (0.9%)	<0.001
**Cardiac death**	153/5144 (3.0%)	130/1478 (8.8%)	23/3666 (0.6%)	<0.001
**Low initial SBP (SBP < 90 mmHg)**				
**Primary endpoint**	44/288 (16.3%)	40/197 (20.3%)	4/91 (4.4%)	<0.001
**Mortality**	44/288 (15.3%)	40/197 (20.3%)	4/91 (4.4%)	<0.001
**Cardiac death**	34/288 (11.8%)	33/197 (16.8%)	1/91 (1.1%)	<0.001
**HFrEF (EF ≤ 40%)**				
**Primary endpoint**	179/3198 (6.0%)	150/1167 (12.9%)	29/2031 (1.4%)	<0.001
**Mortality**	161/3198 (5.0%)	143/1167 (12.3%)	18/2031 (0.9%)	<0.001
**Cardiac death**	132/3198 (4.1%)	119/1167 (10.2%)	13/2031 (0.6%)	<0.001
**HFmrEF (40% < EF < 50%)**				
**Primary endpoint**	24/750 (3.2%)	18/181 (9.9%)	6/569 (1.1%)	<0.001
**Mortality**	23/750 (3.1%)	18/181 (9.9%)	5/569 (0.9%)	<0.001
**Cardiac death**	16/750 (2.1%)	12/181 (6.6%)	4/569 (0.7%)	<0.001
**HFpEF (EF ≥ 50%)**				
**Primary endpoint**	41/1284 (3.2%)	29/266 (10.9%)	12/1018 (1.2%)	<0.001
**Mortality**	39/1284 (3.0%)	29/266 (10.9%)	10/1018 (1.0%)	<0.001
**Cardiac death**	22/1284 (7.1%)	19/266 (7.1%)	3/1018 (0.3%)	<0.001

**Table 3:** Independent predictors of in-hospital clinical outcomes. BMI, body mass index; LVEF, left ventricular ejection fraction; CRF, chronic renal failure.

	Odds Ratio	95% CI	*p*
**Old age (>70 years old)**	2.877	1.908–4.340	<0.001
**Low BMI (<25 kg/m^2^)**	1.587	1.009–2.498	0.046
**Chronic renal failure**	2.254	1.331–3.816	0.002
**LVEF (≤40%)**	1.715	1.111–2.647	0.015
**Uric Acid > 7 mg/dL**	1.689	1.182–2.413	0.004
**CRP > 0.5 mg/dL**	2.636	1.689–4.112	<0.001
**Renal replacement therapy during admission**	10.657	6.763–16.794	<0.001
**Inotrope usage**	5.459	3.622–8.227	<0.001

**Table 4:** Post-discharge 1-month clinical outcomes. SBP, systolic blood pressure; HFpEF, heart failure with preserved ejection fraction; HFrEF, heart failure with reduced ejection fraction.

	Total	Inotrope Users	Inotrope Non-Users	*p* Value
**1-month mortality**	176/5213 (3.4%)	81/1483 (5.5%)	95/3730 (2.5%)	<0.001
**Subgroup—by initial SBP**				
**Low Initial SBP**	20/244 (8.2%)	14/157 (8.9%)	6/87 (6.9%)	0.582
**Normal Initial SBP**	156/4941 (3.2%)	67/1309 (5.1%)	89/3632 (2.5%)	<0.001
**Subgroup—by LV systolic function**				
**HFrEF**	114/3037 (3.8%)	61/1024 (6.0%)	53/2013 (2.6%)	<0.001
**HRmrEF**	17/727 (2.3%)	5/163 (3.1%)	12/564 (2.1%)	0.484
**HFpEF**	37/1245 (3.0%)	9/237 (3.8%)	28/1008 (2.8%)	0.406

**Table 5:** Independent predictors of post-discharge 1-month mortality. CRP, C-reactive protein.

	Hazard Ratio	95% CI	*p*
**Old age (>70 years old)**	2.809	1.739–4.539	<0.001
**Hyponatremia**	1.572	1.048–2.358	0.029
**Uric Acid > 7 mg/dL**	1.542	1.052–2.260	0.026
**High CRP**	1.817	1.153–2.863	0.010
**Renal replacement therapy during admission**	2.653	1.487–4.734	0.001
**Inotrope usage**	1.891	1.264–2.829	0.002

**Table 6:** Characteristics and outcomes after propensity score matching.

	Inotrope Users (*n* = 989)	Inotrope Non-Users (*n* = 989)	Standardized Mean Difference	*p* Value
**Sex (male)**	566 (57.2%)	583 (58.9%)	3.2%	0.439
**Age (years old)**	65.6 ± 15.2	67.2 ± 14.5	10.7%	0.014
**BMI (kg/m^2^)**	23.1 ± 3.7	23.1 ± 3.7	0.5%	0.732
**LVEF (%)**	34.3 ± 15.0	32.8 ± 12.9	10.5%	0.020
**Risk factors**				
**HTN, *n* (%)**	560 (56.6%)	523 (52.9%)	7.7%	0.095
**DM, *n* (%)**	371 (37.5%)	369 (37.3%)	0.4%	0.926
Smoking, % *	18.8/21.8/59.4	20.4/21.1/58.4	2.1%	0.656
**Previous MI, *n* (%)**	166 (16.8%)	161 (16.3%)	1.4%	0.762
**Previous PCI, *n* (%)**	168 (17.0%)	180 (18.2%)	3.3%	0.479
**Previous CABG, *n* (%)**	68 (6.9%)	64 (6.5%)	1.7%	0.719
**COPD, *n* (%)**	118 (11.9%)	90 (9.1%)	9.1%	0.048
**CRF, *n* (%)**	152 (15.4%)	118 (11.9%)	9.9%	0.031
**Initial SBP**	126 ± 29	133 ± 30	23.7%	<0.001
**Initial DBP**	76 ± 18	81 ± 20	26.3%	<0.001
**Initial HR**	93 ± 24	94 ± 25	4.1%	0.290
**Valve disease, *n* (%)**	160 (16.2%)	150 (15.2%)	2.9%	0.536
**Previous CVA, *n* (%)**	135 (13.7%)	145 (14.7%)	3.0%	0.513
**Atrial fibrillation, *n* (%)**	269 (27.2%)	251 (25.4%)	4.2%	0.358
**Laboratory analysis**				
**WBC (10^9^/L)**	9010±4040	8680 ± 3790	8.4%	0.059
**Hb (g/dL)**	12.4 ± 2.3	12.6 ± 2.3	8.7%	0.036
**Platelet (10^9^/L)**	209 ± 93	210 ± 80	1.2%	0.976
**Na (m Eq/L)**	137± 5	137 ± 5	4.0%	0.368
**Uric acid (mg/dL)**	7.3 ± 2.8	7.1 ± 3.5	6.3%	0.076
**Creatinine (mg/dL)**	1.54 ± 1.48	1.35 ± 1.16	14.3%	0.001
**Glucose (mg/dL)**	163 ± 80	156 ± 75	7.7%	0.029
**CRP (mg/L)**	2.64 ± 4.59	2.26 ± 3.86	9.0%	0.044
**Clinical events**				
**In-hospital adverse outcomes**	7.9% (78/989)	1.9% (19/989)	NA	<0.001
**1-month mortality**	4.2% (39/921)	2.6% (25/974)	NA	0.045

BMI, body mass index; LVEF, left ventricular ejection fraction; HTN, hypertension; DM, diabetes mellitus; MI, myocardial infarction; PCI, percutaneous coronary intervention; CABG, coronary artery bypass graft surgery; COPD, chronic obstructive pulmonary disease; CRF, chronic renal failure; CVA, cerebrovascular accident; WBC, white blood cell; Hb, hemoglobin; Na; sodium; BUN, blood urea nitrogen; CRP, C-reactive protein; BUN, brain natriuretic peptide; NTproBNP, *n*-terminal brain natriuretic peptide. * smoking: current smoker/ex-smoker/never smoked.

## Supplementary Materials

The following are available online at http://www.mdpi.com/2077-0383/7/10/368/s1, Figure S1: Detailed causes of in-hospital clinical events; Table S1: Specific diseases entities of the main underlying etiology of acute heart failure, ischemic heart disease, Table S2: Concomitant medications of the total population, Table S3: Prescription pattern of inotropes in inotrope users (n = 1703), Table S4: Multivariate analysis for predictors of in-hospital adverse outcomes by subgroup analysis, Table S5: Post-discharge medications, Table S6: Multivariate analysis for predictors of post-discharge 1-month mortality by subgroup analysis, Table S7: Baseline characteristics of patients presenting with low vs. normal initial SBP in the propensity score-matched cohort, Table S8: Characteristics and outcomes after strict propensity score matching; Supplementary Method: The definition of the causes of death.
