# Supplementary material for: Effects of Widespread Inotrope Use in Acute Heart Failure Patients
Source: J Clin Med. 2018 Oct 18;7(10):368. doi: 10.3390/jcm7100368 (PMC6210304; doi:10.3390/jcm7100368)
Supplement: Supplementary file 1 [file jcm-07-00368-s001.pdf]

Supplementary Materials: Effects of Widespread Inotrope Use in Acute Heart Failure Patients

Jeehoon Kang, Hyun-Jai Cho, Hae-Young Lee, Sangjun Lee, Sue K. Park, Sang Eun Lee, Jae-Joong Kim, Eun-Seok Jeon, Shung Chull Chae, Sang Hong Baek, Seok-Min Kang, Dong-Ju Choi, Byung-Su Yoo, Kye Hun Kim, Myeong-Chan Cho and Byung-Hee Oh

1. Supplementary Methods.

In-hospital mortalities and the causes of death were adjudicated by an independent event committee. This adjudication form was referenced by the adjudication of death of typical 3-phase randomized control trials such as RELAX and RELAX-AHF-2 trial. The Definition of the Causes of Death are as follows:

1. **Death due to Heart failure (HF) progression:** Death occurring in the context of clinically worsening symptoms and/or signs of heart failure. New or worsening signs and/or symptoms of congestive HF may include any of the following:

- New or increasing symptoms and/or signs of HF requiring the initiation of, or an increase in, treatment directed at HF or occurring in a patient already receiving maximal therapy for HF
- HF symptoms or signs requiring continuous intravenous therapy or oxygen administration
- Cardiogenic shock, manifested as clinical signs and symptoms of hypoperfusion felt to be secondary to cardiac dysfunction, and not occurring in the context of an acute myocardial infarction or as the consequence of a primary arrhythmic event

Patients who are hospitalized and are being actively treated for HF and who have an arrhythmia as the terminal event will be classified as having a HF-related death.

2. **Sudden cardiac death:** Death that occurs unexpectedly in a previously stable patie nt will be adjudicated as being sudden cardiac death. The death can be further catego rized as witnessed or unwitnessed:

<u>Witnessed sudden cardiac death:</u>

- Witnessed within 60 minutes of the onset of new or worsening cardiac symptoms
- Witnessed and attributed to an identified arrhythmia (e.g., captured on an electrocardiographic (ECG) recording or witnessed on a monitor by either a medic or paramedic)
- Subjects unsuccessfully resuscitated from cardiac arrest or successfully resuscitated from cardiac arrest but who die without identification of a non-cardiac etiology

Note that if a witnessed sudden cardiac death occurs as a complication of another primary cardiac process, eg, cardiogenic shock or acute myocardial infarction, the primary process should be adjudicated as the cause of death.

<u>Unwitnessed sudden cardiac death:</u>

An unwitnessed death is one that occurs in a patient who when last seen alive within an observation period of 72 hours:

- Did not manifest another life-threatening non-cardiac disease (e.g., infectious, metabolic disorders); and/or
- Did not reveal a cause other than cardiovascular (e.g., trauma) at the scene of death; and/or
- Death was ruled cardiovascular in cause on an autopsy report or death certificate, and occurred in the absence of pre-existing circulatory failure or other modes of death.

3. **Death due to Acute Coronary Syndrome:** Death occurring up to 14 days after a d

ocumented acute coronary syndrome and/or acute myocardial infarction and where there is no conclusive evidence of another cause of death. If death occurs before biochemical confirmation of myocardial necrosis can be obtained, adjudication should be based on clinical presentation and ECG evidence.

4. **Death due to Cerebrovascular Event:** Death occurring up to 30 days after a suspected or confirmed stroke (ischemic stroke or hemorrhagic stroke) based on clinical signs and symptoms as well as neuroimaging and/or autopsy, and where there is no conclusive evidence of another cause of death.

5. **Death due to Other Cardiovascular Causes:** Death must be due to a documented cardiovascular cause not included in the above categories (e.g., peripheral vascular disease, systemic embolus, pulmonary embolism, cardiac procedure complication, and so on)

6. **Death due to Non-Cardiovascular cause:** Non-cardiovascular death is defined as any death not covered by cardiac death or vascular death

2. Supplementary Tables

Table S1. Specific diseases entity of the main underlying etiology of acute heart failure, ischemic heart disease.

Ischemic heart disease*	Total population (*n* = 2096)
Ischemic cardiomyopathy	650 (31.0%)
Acute coronary syndrome	1202 (57.3%)
-STEMI	382 (18.2%)
-NSTEMI	578 (27.6%)
-Unstable Angina	244 (11.6%)
-Miscellaneous	242 (11.5%)

* Ischemic heart disease was defined as a proven coroanry artery stenosis that can clinically explain the pathophysology of heart failure, with no other plausible explanation.

Table S2. Concomitant medications of the total population.

	Total population (*n* = 5471)	Inotrope users (*n* = 1703)	Inotrope non-users (*n* = 3768)	*p* value
**Medication during admission**				
ACE inhibitors	2119 (38.7%)	696 (40.9%)	1423 (37.8%)	0.029
ARB	2972 (54.3%)	1017 (59.7%)	1955 (51.9%)	<0.001
Statin	2549 (46.6%)	827 (48.6%)	1722 (45.7%)	0.050
Beta blocker	2271 (41.5%)	830 (48.7%)	1441 (38.2%)	<0.001
Ivabradine	6 (0.1%)	5 (0.3%)	1 (<0.1%)	0.013
Oral nitrate	3100 (56.7%)	1092 (64.1%)	2008 (53.3%)	<0.001
Loop diuretics	5007 (91.5%)	1614 (94.8%)	3393 (90.0%)	<0.001
Thiazides	748 (13.7%)	280 (16.4%)	468 (12.4%)	<0.001
Amiodarone	846 (15.5%)	497 (29.2%)	349 (9.3%)	<0.001
Digoxin	1836 (33.6%)	663 (38.9%)	1173 (31.1%)	<0.001
Aspirin	3560 (65.1%)	1131 (66.4%)	2429 (64.5%)	0.162
Warfarin	1713 (31.3%)	536 (31.5%)	1177 (31.2%)	0.861
Heparin	2543 (46.5%)	1033 (60.7%)	1510 (40.1%)	<0.001
IV vasodilators	2270 (41.5%)	844 (49.6%)	1426 (37.8%)	<0.001

IV diuretics	4108 (75.1%)	1430 (84.0%)	2678 (71.1%)	<0.001
**Assisting device**				
-IABP	195 (3.6%)	173 (10.2%)	22 (0.6%)	<0.001
-ECMO	151 (2.8%)	145 (8.5%)	6 (0.2%)	<0.001
-LVAD	3 (0.1%)	3 (0.2%)	0 (0%)	0.030
-CRT	47 (0.9%)	30 (1.8%)	17 (0.5%)	<0.001

ACE, Angiotensin-converting enzyme; ARB, Angiotensin receptor blocker; IV, intravenous; IABP, Intra-aortic balloon pump; ECMO, Extracorporeal membrane oxygenation; LVAD, left ventricular assist device; CRT, Cardiac resynchronization therapy.

Table S3. Prescription pattern of inotropes in inotrope users (n = 1703).

	**Inotrope users (n = 1703)**
Dopamine	961 (56.4%)
Dobutamine	1245 (73.1%)
Norepinephrine	521 (30.6%)
Milrinone	133 (7.8%)
Number of Inotropic agent per patient	1.68 ± 0.88
-Single inotropic agent	942 (55.3%)
-Combination of inotropic agents	761 (44.7%)

Table S4. Multivariate analysis for predictors of in hospital adverse outcomes by subgroup analysis.

	**Low Initial SBP (< 90 mmHg)**			**Normal Initial SBP (≥ 90 mmHg)**		
	**OR**	**95% CI**	***p***	**OR**	**95% CI**	***p***
Old age (> 70 years old)	-	-	-	2.886	1.840–4.528	<0.001
Low BMI (< 25 kg/m^2)	-	-	-	1.807	1.099–2.973	0.020
Chronic renal failure	-	-	-	2.762	1.547–4.929	0.001
Uric Acid >7 mg/dL	-	-	-	1.604	1.095–2.348	0.015
High CRP	-	-	-	2.675	1.665–4.297	<0.001
Low LVEF (< 40%)	-	-	-	1.737	1.093–2.760	0.020
Renal Replacement therapy	13.572	3.648–50.497	<0.001	10.762	6.515–17.778	<0.001
Inotrope usage	2.694	0.548–13.258	0.223	5.931	3.864–9.104	<0.001
	HFrEF (LVEF ≤ 40%)			HFpEF (LVEF ≥50%)		
	**OR**	**95% CI**	***p***	**OR**	**95% CI**	**p**
Old age (> 70 years old)	3.310	2.087–5.249	<0.001	-	-	-
Chronic renal failure	2.064	1.133–3.757	0.018	-	-	-
Uric Acid >7 mg/dL	1.671	1.110–2.514	0.014	-	-	-
High CRP	2.487	1.514–4.086	<0.001	4.576	1.059–19.997	0.042
Renal Replacement therapy	8.964	5.246–15.316	<0.001	37.513	10.351–135.955	<0.001
Inotrope usage	5.699	3.529–9.205	<0.001	4.374	1.476–12.962	0.008

SBP, systolic blood pressure; HFpEF, heart failure with preserved ejection fraction; HFrEF, heart failure with reduced ejection fraction; OR, odds ratio; HR, Hazard ratio; CRP, C-reactive protein; BMI, body mass index; LVEF, left ventricular ejection fraction.

Table S5. Post-discharge medications.

Post-Discharge Medication	Inotrope Users (*n* = 1402)	Inotrope Non-Users (*n* = 3635)	*p* Value
ACE inhibitors	369 (30.1%)	1011 (32.5%)	0.127
ARB	444 (36.2%)	1559 (50.1%)	<0.001
Statin	592 (48.3%)	1469 (47.2%)	0.526
Beta blocker	643 (52.4%)	2001 (64.3%)	<0.001
Ivabradine	5 (0.4%)	4 (0.1%)	0.129
Oral nitrate	257 (21.0%)	747 (24.0%)	0.032
Loop diuretics	875 (71.4%)	2424 (77.9%)	<0.001
Thiazides	161 (13.1%)	374 (12.0%)	0.317
Amiodarone	128 (10.4%)	199 (6.4%)	<0.001
Digoxin	355 (29.0%)	881 (28.3%)	0.676
Aspirin	694 (56.6%)	1789 (57.5%)	0.590
Warfarin	391 (31.9%)	1005 (32.3%)	0.794

ACE, Angiotensin-converting enzyme; ARB, Angiotensin receptor blocker.

Table S6. Multivariate analysis for predictors of post-discharge 1-month mortality by subgroup analysis.

	**Low Initial SBP (< 90 mmHg)**			**Normal Initial SBP (≥ 90 mmHg)**		
	**HR**	**95% CI**	***p***	**HR**	**95% CI**	***p***
Old age (> 70 years old)	11.091	1.910–64.397	0.007	3.600	0.953–13.599	0.059
Hyponatremia	-	-	-	2.753	1.015–7.471	0.047
Renal Replacement therapy	24.114	2.763–210.481	0.004	2.165	1.147–4.090	0.017
Inotrope usage	0.644	0.110–3.752	0.624	3.584	1.280–10.037	0.015
	HFrEF (LVEF ≤ 40%)			HFpEF (LVEF ≥ 50%)		
	**HR**	**95% CI**	***p***	**HR**	**95% CI**	***p***
LV ejection fraction (per 1%)	0.890	0.810–0.978	0.016	-	-	-
Inotrope usage	1.152	0.288–4.616	0.841	54.666	3.479–858.978	0.004

SBP, systolic blood pressure; LV, left ventricle; HFpEF, heart failure with preserved ejection fraction; HFrEF, heart failure with reduced ejection fraction; OR, odds ratio; HR, Hazard ratio.

Table S7. Baseline characteristics of patients presenting with low vs normal initial SBP in the propensity score matched cohort.

	**Low initial SBP (< 90 mmHg) (*n* = 114)**	**Normal initial SBP (≥ 90 mmHg) (*n* = 1864)**	***p* value**
Sex (male)	61 (53.5%)	1088 (58.4%)	0.307
Age (years old)	63.6±15.0	66.6±14.8	0.034
BMI (kg/m^2)	22.3±3.6	23.2±3.7	0.015
LVEF (%)	33.5±14.0	33.8±14.6	0.871
Risk factors			
HTN, n (%)	36 (31.6%)	1047 (56.2%)	<0.001
DM, n (%)	25 (21.9%)	715 (38.4%)	<0.001
Smoking, %*	21.9/24.6/53.5	19.5 / 21.3 / 59.2	0.482
Previous MI, n (%)	18 (15.8%)	309 (16.6%)	0.826
Previous PCI, n (%)	17 (14.9%)	331 (17.8%)	0.439
Previous CABG, n (%)	9 (7.9%)	123 (6.6%)	0.590
COPD, n (%)	10 (8.8%)	198 (10.6%)	0.532
CRF, n (%)	13 (11.4%)	257 (13.8%)	0.472

Initial SBP	79±10	133±28	<0.001
Initial DBP	51±9	80±18	<0.001
Initial HR	86±29	94±24	0.006
Valve disease, n (%)	10 (8.8%)	300 (16.1%)	0.037
Previous CVA, n (%)	21 (18.4%)	259 (13.9%)	0.179
Atrial fibrillation, n (%)	83 (72.8%)	1375 (73.8%)	0.821
Laboratory analysis			
WBC (10^9/L)	9230±4430	8820±3890	0.337
Hb (g/dL)	12.2±2.7	12.5±2.3	0.141
Platelet (10^9/L)	210±78	209±87	0.995
Na (mEq/L)	136±5	137±5	0.010
Uric acid (mg/dL)	7.7±3.2	7.1±3.2	0.055
Creatinine (mg/dL)	1.67±1.98	1.44±1.28	0.211
CRP (mg/L)	2.69±3.70	2.43±4.28	0.537
Clinical events			
In-hospital adverse outcomes	9.6% (11/114)	4.6% (86/1864)	0.016
1 month mortality	1.9% (2/104)	3.5% (62/1791)	0.398

BMI, body mass index; LVEF, left ventricular ejection fraction; HTN, hypertension; DM, diabetes mellitus; MI, myocardial infarction; PCI, percutaneous coronary intervention; CABG, coronary artery bypass graft surgery; COPD, chronic obstructive pulmonary disease; CRF, chronic renal failure; CVA, cerebrovascular accident; WBC, white blood cell; Hb, hemoglobin; Na; sodium; BUN, blood urea nitrogen; CRP, C-reactive protein; BUN, brain natriuretic peptide; NTproBNP, n-terminal Brain natriuretic peptide. * smoking: current smoker / ex- smoker / never smoker.

Table S8. Characteristics and outcomes after strict propensity score matching.

	**Inotrope Users (*n* = 238)**	**Inotrope Non-Users (*n* = 238)**		
	**Mean (SD)**	**Mean (SD)**	**SMD**	***p* Value**
Age (years old)	68.8 (13.5)	69.1 (13.0)	0.017	0.85
BMI (kg/m²)	23.4 (3.9)	23.6 (3.9)	0.043	0.64
Initial SBP (mmHg)	132.1 (30.9)	136.0 (29.3)	0.129	0.16
Initial DBP (mmHg)	76.1 (18.5)	78.9 (19.3)	0.148	0.11
Initial HR (bpm)	95.3 (23.8)	96.1 (23.8)	0.032	0.73
BSA (m²)	1.7 (0.2)	1.7 (0.2)	0.010	0.91
Pulse (beat/minute)	95.3 (23.8)	96.1 (23.8)	0.032	0.73
LVEF (%)	33.5 (13.9)	34.5 (14.5)	0.069	0.45
Laboratory analysis				
CK-MB	12.4 (28.7)	8.6 (20.2)	0.152	0.10
WBC (10^9/L)	9,576.6 (4,487.7)	9,235.2 (3,662.9)	0.083	0.36
Hb (g/dL)	12.4 (2.4)	12.4 (2.2)	0.021	0.82
Platelet (10^9/L)	217.3 (87.2)	224.7 (78.7)	0.089	0.33
Na (mEq/L)	137.8 (4.6)	137.8 (4.5)	0.015	0.87
K (mEq/L)	4.4 (0.7)	4.4 (0.7)	0.033	0.72
Uric acid (mg/dL)	7.4 (2.7)	6.9 (3.9)	0.132	0.15
Creatinine (mg/dL)	1.5 (1.5)	1.4 (1.3)	0.043	0.64
Glucose (mg/dL)	182.6 (93.6)	179.8 (86.9)	0.031	0.74
RDW (%)	14.6 (2.3)	14.4 (1.8)	0.097	0.29
HbA1C (%)	6.7 (1.3)	6.8 (1.3)	0.051	0.58
Total cholesterol (mg/dL)	161.7 (44.6)	162.8 (45.0)	0.024	0.79
TG (mg/dL)	102.8 (74.2)	105.5 (66.1)	0.037	0.68
HDL (mg/dL)	39.1 (14.5)	40.5 (12.8)	0.097	0.29
Albumin (g/dL)	3.6 (0.5)	3.7 (0.5)	0.113	0.22
BUN (mg/dL)	25.2 (15.3)	27.7 (16.9)	0.155	0.09
cTNI (ng/mL)	2.8 (7.6)	2.0 (8.2)	0.104	0.26
CRP (mg/L)	2.8 (4.8)	2.4 (3.9)	0.090	0.33
	**N (%)**	**N (%)**	**SMD**	***p***
Sex (male)	140 (58.8)	136 (57.1)	0.034	0.78
HTN	155 (65.1)	157 (66.0)	0.018	0.92
DM	126 (52.9)	131 (55.0)	0.042	0.71
Current smoking	64 (26.9)	57 (23.9)	0.116	0.45
Previous MI	37 (15.5)	41 (17.2)	0.045	0.71

Previous PCI	44 (18.5)	37 (15.5)	0.078	0.46
Previous CABG	10 (4.2)	12 (5.0)	0.040	0.83
COPD	30 (12.6)	24 (10.1)	0.080	0.47
CRF	28 (11.8)	27 (11.3)	0.013	1.00
Valve disease	30 (12.6)	20 (8.4)	0.137	0.18
Previous CVA	32 (13.4)	36 (15.1)	0.048	0.69
Atrial fibrillation	45 (18.9)	50 (21.0)	0.053	0.65
NYHA IV	153 (64.3)	153 (64.3)	0.082	0.67
Renal transplantation	11 (4.6)	19 (8.0)	0.139	0.19
Vasodilater medication	63 (26.5)	52 (21.8)	0.108	0.28
BNP≥500 or NTproBNP ≥ 1000 (pg/mL)	199 (83.6)	200 (84.0)	0.011	1.00
Clinical events				
in-hospital adverse events	19 (8.0%)	5 (2.1%)	NA	<0.01
1-month mortality	7 (3.2%)	4 (1.7%)	NA	0.37

BMI, body mass index; SBP, systolic blood pressure; DBP, diastolic blood pressure; HR, heart rate; BSA, body surface area; LVEF, left ventricular ejection fraction; CK-MB, creatine kinase-muscle/brain; WBC, white blood cell; Hb, hemoglobin; Na; sodium; K, potassium; RDW, red cell distribution width; TG, triglyceride; HDL, high-density lipoproteins; BUN, blood urea nitrogen; cTNI, cardiac troponin-I; CRP, C-reactive protein; HTN, hypertension; DM, diabetes mellitus; MI, myocardial infarction; PCI, percutaneous coronary intervention; CABG, coronary artery bypass graft surgery; COPD, chronic obstructive pulmonary disease; CRF, chronic renal failure; CVA, cerebrovascular accident; NYHA, New York Heart Association; NTproBNP, n-terminal Brain natriuretic peptide; SMD, standardized mean difference.

3. Supplementary Figure

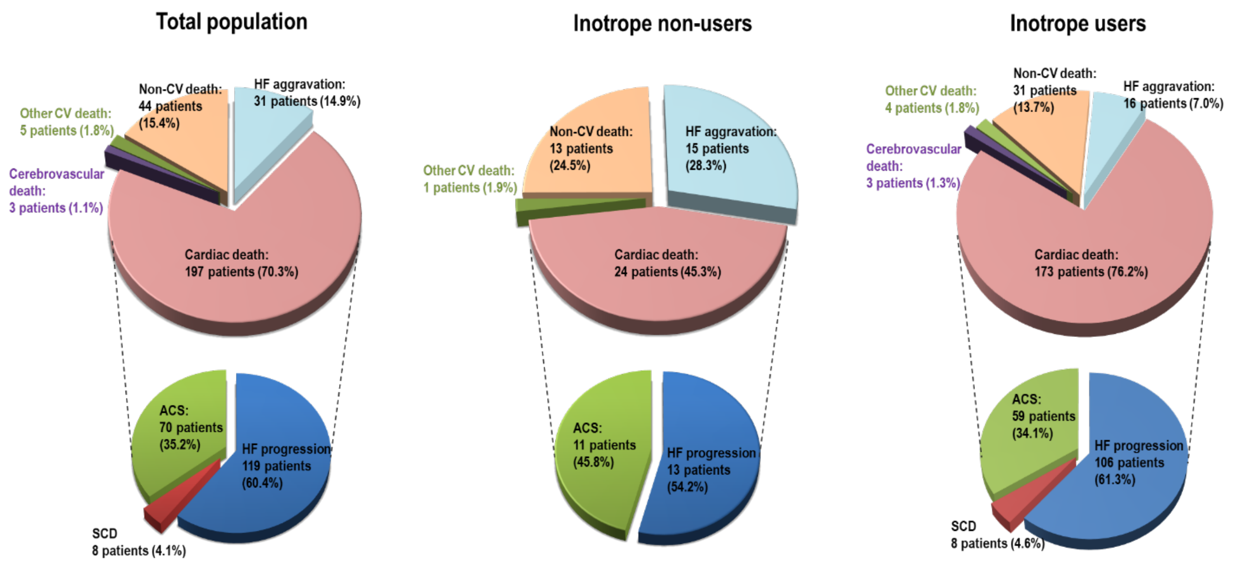

Figure S1. Detailed cause of in-hospital clinical events.

In-hospital clinical events occurred in 280 (5.1%) of the cases during admission. Cardiac death occupied the majority of in-hospital clinical events (70.3%), and the most common cause of cardiac death was HF aggravation (60.4%). In subgroup analysis, cardiac death was more common in inotrope users (45.3% [24/53] vs 76.2% [173/227], $p < 0.001$), while non-cardiovascular death events were more common in inotrope non-users (24.5% [13/53] vs 13.7% [31/227], p = 0.050). All 8 cases (4.1%) of sudden cardiac death events occurred in inotrope users.
